# Should patients with diabetes be encouraged to integrate social media into their care plan?

**DOI:** 10.4155/fsoa-2018-0021

**Published:** 2018-07-31

**Authors:** Arun R Nelakurthi, Angela M Pinto, Curtiss B Cook, Lynne Jones, Mary Boyle, Jieping Ye, Theodoros Lappas, Jingrui He

**Affiliations:** 1 School of Computing, Informatics, & Decision Systems Engineering, Arizona State University, Tempe, AZ, 85281, USA; 2 Department of Psychology, Baruch College, City University of New York, New York, NY, 10010, USA; 3 Division of Endocrinology, Mayo Clinic Arizona, Scottsdale, AZ, 85259, USA; 4 Department of Computational Medicine & Bioinformatics, University of Michigan, Ann Arbor, MI, 48109, USA; 5 School of Business, Stevens Institute of Technology, Hoboken, NJ, 07030, USA

**Keywords:** diabetes mellitus, endocrinology, obesity, personalized medicine, social media, social networking

## Abstract

**Aim:**

To evaluate the use of social media of individuals with diabetes mellitus (DM).

**Materials & methods:**

Both web-based and in-clinic surveys were collected from individuals with DM. Descriptive and correlation analyses were employed to evaluate respondents’ diabetes-specific social networking site behaviors.

**Results:**

Forty-five patients with DM completed the web-based survey and 167, the clinic-based survey, of whom only 40 visited diabetes-specific social networking sites. Analysis of online survey data indicated that self-reported adherence to lifestyle recommendations was significantly correlated (p < 0.01) with visiting the sites. Clinic-based survey data found that patients who reported using DM-specific web sites monitored home glucose values more often and had better compliance with insulin administration (both p < 0.05) compared with nonusers.

**Conclusion:**

This study provides insight into why individuals visit DM-specific social networking sites. Certain self-management behaviors may improve as a result of visiting these sites. Further work is needed to explore how to leverage social media technology to assist patients with the management of DM.

## Lay abstract

Social media has gained prominence as a source for healthcare information. This study used both online surveys and surveys of clinic patients with diabetes to understand why patients with diabetes use DM-specific social networking sites. The authors found that patients go to these resources to both offer and receive encouragement for their health condition. Visiting DM-specific social networking sites influenced more positive diabetes care behaviors, such as adhering to diet and exercise, glucose monitoring and insulin use. Further research is needed to understand how to incorporate social media into the care of diabetes.

Social media is becoming an important resource for healthcare information [1]. Based on a survey of 2008 US adults, 87% used the internet to access information online [2]. Widespread access to the internet has made it relatively easy for people to go online to seek answers to their health-related questions. It is estimated that in the USA, health information is sought online by 79% of internet users. Moreover, the percentage of users that use the internet as a source of health information rapidly increased from 38% of users in 1998 to 79% in 2011 [3,4]. In addition, many patients use the internet to find and join communities of individuals with similar health conditions to share information, as well as to provide and receive support for healthcare management [5,6].

Diabetes mellitus (DM) is a chronic illness that can be effectively managed through physical activity, healthy dietary habits and the appropriate and timely use of pharmacotherapies to lower blood glucose levels [7]. Research has demonstrated that online social support programs like healthcare forums and social media websites (e.g., Facebook and Twitter) can help patients with Type 1 DM on insulin pump therapy gain knowledge about their disease and cope better with their daily management routine [8]. Such platforms can allow patients to share personal clinical information, request diabetes-specific advice and receive the emotional support that they need for diabetes management and self-care [9].

Most of the research on the influence of social media on DM care has focused on widely used platforms without a clear focus on a specific health condition [8–10]. Research has shown that such generic social media platforms have lots of promotional activity and personal data collection but no checks for authenticity [8]. On the other hand, most of the DM-specific social networking sites are moderated and enforce measures on patient privacy [9]. Often moderated platforms ensure authenticity and correctness in the information delivered to users. Another study of preferences for online DM support found that adults generally preferred professionally moderated discussions [11]. Nonetheless, there remains a paucity of data on how patients with DM use social media or how it may impact their behaviors. Therefore, internet- and clinic-based surveys were conducted of patients with DM to assess the reason individuals visited DM-specific social networking sites and what impact such use had on specific self-management behaviors.

## Materials & methods

### Data collection

Information regarding DM was collected through surveys. As part of the survey, participants were asked to answer questions broadly classified as: demographic information; educational level; marital status; diabetes-specific information (e.g., diabetes diagnosis, most recent hemoglobin A1c [HbA1c] value); nature and frequency of diabetes-specific social networking site usage; dietary habits and diabetes self-care activities and reasons for using the site [12]. The surveys were collected in two modalities: anonymous, web-based survey and from patients at an in-clinic setting. The diabetes-specific social networking site questions are provided in the Supplementary Material.

Both the web-based and clinic surveys were convenience samples. The web-based survey data were collected through a self-administered Qualtrics platform, advertised through DM-specific social networking sites (see Supplementary Material for list of sites). Participation was anonymous and required individuals to be at least 18 years old. The clinic-based survey was offered to all patients with DM returning for a follow-up appointment to an outpatient academic endocrinology practice in the southwest USA. Once individuals read a brief consent document and agreed to participate, they proceeded with the self-completed paper survey. No compensation was offered for participation. Questions on the clinic survey were identical to the web-based version, except formatting was adjusted to accommodate the flow of the outpatient setting (Supplementary Material). Additionally, the clinic-based survey incorporated chart review to obtain accurate information on medications, most recent HbA1c value and body mass index. A question on self-reported income range was added as well. The study was approved by the Institutional Review Boards of the participating institutions.

### Data analysis

The demographic, DM-specific and social media usage information were analyzed using descriptive statistics. Associations between the use of DM-specific social networking website and DM-related behaviors were analyzed using correlation analysis with Pearson's correlation coefficient for analysis of the online survey [13]. The responses to the survey questions on DM-specific social networking sites usage contain both ordinal and categorical Likert scale-based responses [14]. For convenience, the categorical responses were converted into a numerical scale with order and rank preserved. Questions and their responses were assumed to be independent of each other. For the clinic-based surveys, differences between social media users and nonusers were evaluated via *t*-test for continuous variables and χ^2^ for categorical variables.

## Results

### Respondent characteristics

A total of 45 participants from the USA (n = 31) and UK (n = 14) combined submitted their responses to the web-based survey (Table 1). Mean (SD) age was 57 (14) years, most were women, identified themselves as white, had attended college and were not working. The participant pool consisted of a balanced mix of patients who reported they had Type 1 DM and Type 2 DM. The mean (SD) self-reported HbA1c was 7.0 (2.2)%.

**Table 1.** **Demographic information of 45 participants participating in online survey.**

**Characteristic**	**Sub-characteristic**	**Mean (SD) or n (%)**
Age, years		57 (14)

Gender	Women	30 (67%)

	Men	15 (33%)

White race		45 (100%)

Diabetes duration, years		17 (14)

Diabetes diagnosis	Type 1	21 (47%)

	Type 2	22 (49%)

	Other	2 (4%)

Prescribed insulin for diabetes		29 (64%)

Hemoglobin A1c,%		7.0% (2.2%)

Education	Did not complete high school	1 (2%)

	Completed high school	4 (9%)

	Some college or vocational training	17 (38%)

	Four-year college or higher	23 (51%)

Employment status	Working full or part time	12 (27%)

	Unemployed/retired/student	33 (73%)

SD: Standard deviation.

Between December 2016 and March 2017, 167 patients with DM participated in the clinic survey (Table 2). Of these, 40 (31%) indicated they used DM-specific social networking sites. Social media users were significantly younger than nonusers (p < 0.01). Additionally, in this clinic sample, a significant difference in the distribution of diabetes type was detected (p = 0.034) between users and nonusers. More patients with Type 2 DM than Type 1 DM used DM-specific social networking sites. However, the proportion of patients with Type 1 DM who were users (33%) was higher than nonusers (14%), while the percentage of those with Type 2 DM who were nonusers (82%) was greater than users (65%). Marital status was also different (p = 0.017), with a greater proportion of users indicating they were not married, while more nonusers reported they were married. The distribution of employment status was dissimilar (p = 0.016), with more social media users working, while a greater proportion of nonusers were not currently working. No differences in race/ethnicity, self-reported diabetes duration, insulin use, HbA1c, body mass index, education or income level were found.

**Table 2.** **Demographic information 167 diabetes patients completing clinic-based questionnaire on use of social media.**

**Characteristic**	**Social media use**		

	**Yes n = 40**	**No n = 127**	**p-value**
Age, years	54 (16)	64 (12)	<.01

Gender	–	–	0.36

Women	19 (48%)	50 (39%)	–

Men	21 (52%)	77 (61%)	–

White race	36 (90%)	112 (88%)	0.75

Diabetes duration, years	13 (11)	16 (13)	0.23

Diabetes diagnosis	–	–	0.034

Type 1	13 (33%)	18 (14%)	–

Type 2	26 (65%)	104 (82%)	–

Other	1 (2%)	5 (4%)	–

Prescribed insulin for diabetes	35 (87%)	98 (77%)	0.16

Body mass index, (kg/m^2^)	30.8 (5.5)	31.2 (6.7)	0.69

Hemoglobin A1c,%	7.7 (1.6)	7.6 (1.6)	0.66

Marital status	–	–	0.017

Married	18 (45%)	84 (66%)	–

Not married	22 (55%)	43 (34%)	–

Education	–	–	0.13

Did not complete high school	7 (17%)	17 (13%)	–

Completed high school	2 (5%)	27 (21%)	–

Some college or vocational training	14 (35%)	38 (30%)	–

Four-year college or higher	17 (43%)	45 (35%)	–

Employment Status	–	–	0.016

Working full or part	21 (52%)	40 (31%)	–

Unemployed/retired/student	19 (48%)	87 (69%)	–

Income, thousands of US dollars	–	–	0.53

<55	13 (32%)	46 (36%)	–

55–99	11 (27%)	28 (22%)	–

100–150	7 (18%)	20 (16%)	–

>150	6 (15%)	12 (9%)	–

Declined to respond	3 (8%)	21 (17%)	–

### Website usage

**Figure 1.**
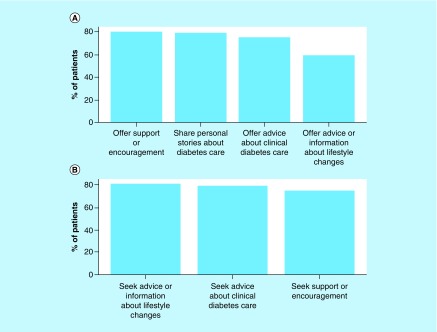
**Top reasons for social media website usage.** Diabetes mellitus survey participants who login to the social media websites at least two to three times a week to **(A)** post information and **(B)** seek advice.

The authors were interested in the distribution of why survey respondents went to DM-specific social networking sites. It was assumed that the two reasons social media users may go to sites would be to either offer advice or support or to seek advice or support. For this analysis, data from the online and clinic-based surveys were combined and considered only individuals who frequently (defined as at least 2–3 times a week) logged into the DM-specific social networking sites and analyzed the frequency of reasons for both offering and seeking information (derived from Question 15). The two most common reasons respondents indicated they would be moderately to extremely likely to visit the website was to offer support or encouragement to other individuals with DM or to share personal experiences (Figure 1A). For individuals visiting the websites for personal reasons, the most common reason was to seek support or encouragement from individuals with DM, to seek advice about clinical diabetes care and obtain advice about lifestyle changes (Figure 1B).

### Behaviors associated with use of DM-specific social networking sites

**Figure 2.**
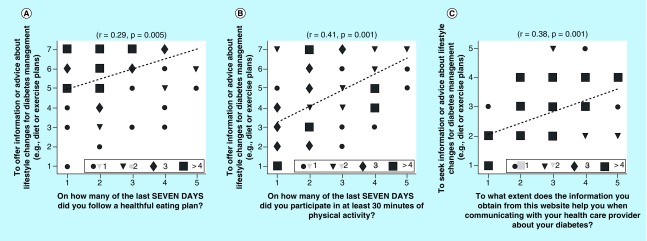
**Correlation results between information or advice offering and seeking behavior and other behavioral traits of diabetes mellitus survey participants.** Panel **(A)** shows correlation results from respondents who usedsocial media to provide advice about diabetes vs. their own self-reported eating habits. Panel **(B)** shows the analysis of respondents who used social media to provide advice about diabetes vs. their own self-reported exercise habits. Panel **(C)** illustrates the correlation between using social media to obtain advice about diabetes vs. tendency to communicate with their health care provider. Data is derived from the web-based survey. The correlation parameters r and p-value are shown in the subfigure title. The symbols represent number of respondents (circle = one response, triangle = two, diamond = three and square = four).

Next, we separately examined self-reported behaviors reported by persons who completed the web-based survey data and those who completed the clinic-based survey. Looking at the data from the web-based survey first, significant positive correlations were observed between respondents who offered advice on social media versus their own self-reported eating (Figure 2A) and exercise habits (Figure 2B). Furthermore, using the website to obtain information on lifestyle changes for diabetes management was positively associated with following that advice (Figure 2c).

Clinic-based surveys did reveal some significant differences between DM-specific social networking siteusers and nonusers. Users had a slight but significant increased frequency in the number of days per week where they reported checking their glucose. DM-specific social networking site users reported checking their glucose on average 7 (1) days per week while nonusers checked 6 (2) days per week (p = 0.047). Self-reported insulin therapy compliance was also slightly but significantly (p = 0.014) higher among users of social media, with users taking insulin 7 (1) days per week and nonusers 6 (2) days per week. No differences in other behaviors, such as dietary and exercise compliance or compliance with oral medications were detected.

### Compliance, healthcare provider communication & social media use

When combining web and clinic based surveys (n = 85), data showed that 57/85 (69%) of respondents were moderately to extremely likely to follow the advice they received from the website about lifestyle changes for DM management and 54/85 (65%) were moderately to extremely likely to follow the advice received from the website about clinical DM care (e.g., blood sugar monitoring, medications, etc.). Moreover, the information obtained from the websites helped about 52/85 (63%) of DM survey participants in communicating with their healthcare provider about their DM.

## Discussion

Social media has now become an important source of healthcare information for patients and their caregivers. Patients use online resources to join the community of patients with similar diagnoses to engage, share information on management of their health conditions and provide and receive support for themselves. Previous studies have shown that social media websites can help patients gain knowledge about DM and information to cope better with their daily management routine. However, little is known about the reasons they may visit these sites or if visiting these sites somehow positively impacts diabetes self-management behaviors.

In the current study, we assessed DM-specific social networking site utilization to gain an insight into how patients employ these resources and to determine if there was any impact on self-management behaviors. Two different approaches were applied. First, an online survey was conducted of individuals while they were actively utilizing the website. This provided information on users only. Second, an assessment of a clinic-based population of patients with DM was conducted. The second method, in addition to providing information on prevalence of DM-specific social networking site usage in a clinic-based population, yielded preliminary data on the characteristics of users and nonusers and differences in self-management behaviors between the groups.

Statistical comparisons are not appropriate between data derived from the websites and the clinic, but some similarities and differences in characteristics of survey participants can be noted in data derived from the two sources of information. For instance, the mean age of participants from the online survey and that of the clinic-based survey was in the 50s. Additionally, website users in both surveys predominately used insulin, were predominately white and had education beyond high school. Those who completed the survey online were comprised mostly of women and reported having balanced mixed of Type 1 and Type 2 DM. In contrast, DM-specific social networking site users who completed the clinic survey had a similar composition of men and women and were mostly patients with Type 2 DM.

The clinic-based survey indicated that the prevalence of DM-specific social networking site use, at least in this population was low – only about a quarter of respondents stated they visited these sites. The clinic survey revealed some differences in users versus nonusers. Social media users were significantly younger, and a greater proportion had Type 1 DM versus nonusers, whereas nonusers had a predominance of Type 2 DM. Additionally, more users reported as not being married and it could be speculated that a lack of family support drove more of the users to seek support on social media sites. Finally, a majority of users were employed, while nonusers were either unemployed, students or retired. It is possible that work-life balance may have driven busier employed individuals to the convenience of social media use.

Beyond simply examining reasons for visiting DM-specific social networking sites, we also sought to evaluate whether utilizing these tools were associated with any specific desirable diabetes behaviors. For instance, when analyzing survey data completed online (i.e., in those actively using the websites), there was a positive correlation between the self-reported frequency of following a healthy dietary plan and performance of physical activity with going to the website to offer advice about these activities. It is likely that individuals going to the sites to offer advice were already successful at self-management and wanted to pass on tips. Analysis of the clinic-based surveys also revealed some positive impacts of DM social media use on reported self-management behaviors. Compared with nonusers, users had a slight but significantly increased compliance with the frequency of weekly glucose monitoring and insulin administration.

Diabetes self-management education and providing individualized strategies to achieve metabolic and lifestyle goals is integral to effective management. To this end, specific standards of care have been developed [15]. What has not been well studied is how contemporary social interactions, such as online social interactions that are common today can be used to assist patients with diabetes to care more effectively for their condition. The data here suggests that such sites have the potential to be used to improve self-management behaviors.

There are limitations to the current study. First, the sample size of website users, both online and clinic-based participants was small. In particular, the low participation in social media use noted in the clinic participants may have precluded detection of differences in other measures, such as HbA1c or body mass index. Furthermore, participants in both the web-based and clinic survey overall had reasonably good glycemic control, as evidenced by the HbA1c (both self-reported from the online survey and measured from the clinic). In the clinic-based survey, social media nonusers had an HbA1c comparable with the users, so the nonusers may have felt that additional advice (such as that gained from social media) may not have been needed to improve glycemic control. Finally, the survey was limited to English speakers only, respondents were largely of white race and behaviors were self-reported.

## Conclusion

Despite the limitations, results of this study provided insight into why individuals visit DM-specific social networking sites. Moreover, certain self-management behaviors, such as self-monitoring of blood glucose and insulin administration may be improved. The results from the in-clinic surveys showed that only a small number of participants use social media for their diabetes management. Further work is needed to explore how to incorporate DM-specific social networking site use into the clinical environment and how to leverage the technology to assist patients with their condition.

## Future work

The role of social media in management of DM remains relatively unexplored. Rather than a convenience sample (such as this study), a randomized control trial, appropriately statistically powered to detect differences in reported self-management behaviors or HbA1c, would be helpful. DM patients (who are currently nonsocial media users) could be randomized to visiting social media sites, with a control group maintaining usual care. Outcomes such as HbA1c and behaviors as measured here could be tracked. Future research that examines the relationship between online posting and diabetes-related self-care behaviors in a longitudinal design would help to clarify the role of website use in diabetes management. Lastly, including a more diverse race/ethnic patient population in future studies is needed.

**Summary points**

- Social media is becoming an important resource for healthcare information.
- There remains a paucity of data on how patients with diabetes mellitus (DM) use social media or how it may impact their behaviors.
- Both internet- and clinic-based surveys were conducted of patients with DM to assess the behaviors of individuals with DM who either offered or sought information on diabetes-specific social media websites.
- Forty-five patients completed the web-based survey and 167 the clinic-based survey, of whom only 40 visited social media sites.
- Respondents most often visited sites to offer support for DM or to seek such support for themselves.
- Analysis of online survey data indicated that self-reported adhering to lifestyle recommendations was significantly correlated (p < 0.01) with visiting the sites.
- Clinic-based survey data found that patients who reported using DM-specific web sites stated they monitored their home glucoses more often and reported better compliance with insulin administration (both p < 0.05) compared with nonusers.
- Study limitations included small sample size, lack of race/ethnic diversity of respondents and self-reported behaviors.
- Further work is needed to explore how to incorporate DM social media use into the clinic environment and how to leverage the technology to assist patients with their condition.

## References

[1] Honigman B (2013). 24 outstanding statistics and figures on how social media has impacted the healthcare industry. *Referral MD*. https://www.getreferralmd.com/2013/09/healthcare-social-media-statistics.

[2] Pew Research Center. (2018). Internet user demographics. http://www.pewinternet.org/data-trend/internet-use/latest-stats/.

[3] Taylor H (2011). The growing influence and use of healthcare information obtained online. *The Harris Poll*.

[4] Fox S, Duggan M (2013). Health Online 2013. *Pew Research Center*.

[5] Ravert RD, Hancock MD, Ingersoll GM (2004). Online forum messages posted by adolescents with Type 1 diabetes. *Diabetes Educ.*.

[6] Farmer AD, Bruckner Holt CE, Cook MJ, Hearing SD (2009). Social networking sites: a novel portal for communication.. *Postgrad. Med. J.*.

[7] Dansinger M (2017). Diabetes Mellitus Type 1 versus 2: symptoms, causes & treatments.. http://www.webmd.com/diabetes/guide/types-of-diabetes-mellitus.

[8] Petrovski G, Zivkovic M, Stratrova SS (2015). Social media and diabetes: can Facebook and Skype improve glucose control in patients with Type 1 diabetes on pump therapy? One-year experience. *Diabetes Care*.

[9] Greene JA, Choudhry NK, Kilabuk E, Shrank WH (2011). Online social networking by patients with diabetes: a qualitative evaluation of communication with Facebook. *J. Gen. Intern. Med.*.

[10] Sparling K, Tenderich A, Warshaw H (2015). Community as part of the prescription: social media in diabetes care. *AJMC*.

[11] Zrebiec J (2005). Internet communities: do they improve coping with diabetes?. *Diabetes Educ.*.

[12] Toobert DJ, Hampson SE, Glasgow RE (2000). The summary of diabetes self-care activities measure. *Diabetes Care*.

[13] Fisher R (1915). Frequency distribution of the values of the correlation coefficient in samples from an indefinitely large population. *Biometrika*.

[14] Likert R (1932). A technique for the measurement of attitudes. *Archives Psychol.*.

[15] American Diabetes Association (2017). Standards of medical care in diabetes. *Diabetes Care*.

