# Supplementary material for: Should patients with diabetes be encouraged to integrate social media into their care plan?
Source: Future Sci OA. 2018 Jul 31;4(7):FSO323. doi: 10.4155/fsoa-2018-0021 (PMC6088271; doi:10.4155/fsoa-2018-0021)
Supplement: Supplementary file 1 [file fsoa-04-323-s1.docx]

1. Which diabetes-specific social networking site do you typically (most often) use?

Community.diabetes.org

Diabeticconnect.com

Diabeticnetwork.com

Diabetes.co.uk

Diabetesdaily.com

Diabetesforum.com

Diabetesisters.org

Diabetes-support.org.uk

Diabetessupport.co.uk

Healingwell.com

Tudiabetes.org

Other:

2. When did you start using this website?

Less than 1 month ago

1-3 months ago 3-6 months ago 6-12 months ago More than 12 months ago

3. On average, how often do you logon to this website?

Less than once a month

Once a month

2-3 times a month

Once a week

2-3 times a week

4-5 times a week

Once a day

More than once a day

4. On average, how often do you post a question to this website asking for information or advice about your diabetes management?

Less than once a month

Once a month

2-3 times a month

Once a week

2-3 times a week

4-5 times a week

Once a day

More than once a day

5. On average, how often do you post information or advice about diabetes management to this website in response to another user’s question or concern?

Less than once a month

Once a month

2-3 times a month

Once a week

2-3 times a week

4-5 times a week

Once a day

More than once a day

6. Please select the option that best describes how likely you are to use this website for the following purposes:

To **offer information about lifestyle changes** for diabetes

Not at all likely

Slightly likely

Moderately likely

Very likely Extremely likely

management (e.g., diet or exercise plans)

1 2 3 4 5

To **offer advice about lifestyle changes** for diabetes management (e,g., diet or

1 2 3 4 5

exercise strategies that worked for you).

To **offer information about clinical diabetes care** (e.g., blood sugar monitoring, medications).

1 2 3 4 5

To **offer advice about clinical diabetes care** (e.g., blood sugar monitoring, medications) based on your experience.

1 2 3 4 5

To **offer support or**

**encouragement** to other users. 1 2 3 4 5

To share personal stories about

your experience with diabetes. 1 2 3 4 5

To **seek information about lifestyle changes** for diabetes management (e.g., diet or exercise plans).

1 2 3 4 5

To **seek advice about lifestyle changes** for diabetes management (e.g., diet or

exercise strategies that worked for others)

1 2 3 4 5

To **seek information about clinical diabetes care** (e.g., blood sugar monitoring, medications).

1 2 3 4 5

To **seek advice about clinical diabetes care** (e.g., blood sugar monitoring, medications) based on others’ experience.

1 2 3 4 5

To **seek support or**

**encouragement** from other users. 1 2 3 4 5

7. How likely are you to follow the advice you receive from this website about lifestyle changes for diabetes management (e.g., specific diet strategies or exercise plans)?

1

Not at all likely

2

Slightly likely

3

Moderately likely

4

Very likely

5

Extremely likely

Not

Applicable

8. How likely are you to follow the advice you receive from this website about clinical diabetes care (e.g., blood sugar monitoring, medications)?

1

Not at all likely

2

Slightly likely

3

Moderately likely

4

Very likely

5

Extremely likely

Not

Applicable

9. To what extent does the information and/or support you obtain from this website help you when communicating with your health care provider about your diabetes?

1

Not at all

2

Slightly

3

Moderately

4

Very much

5

Extremely

Not

Applicable

10. When you have a question about your diabetes care, are you more likely to contact your health care provider or consult this website?

Contact health care provider Consult diabetes-specific social networking site
